# Tree Crowns Segmentation and Classification in Overlapping Orchards Based on Satellite Images and Unsupervised Learning Algorithms

**DOI:** 10.3390/jimaging7110241

**Published:** 2021-11-17

**Authors:** Abdellatif Moussaid, Sanaa El Fkihi, Yahya Zennayi

**Affiliations:** 1 Information Retrieval and Data Analytics Laboratory, ENSIAS, Mohammed V University in Rabat, Rabat 10100, Morocco; sanaa.elfkihi@ensias.um5.ac.ma; 2 Embedded Systems and Artificial Intelligence Department, Moroccan Foundation for Advanced Science Innovation and Research (MAScIR), Rabat 10100, Morocco; y.zennayi@mascir.ma

**Keywords:** tree canopy segmentation, tree canopy classification, unsupervised learning, satellite images, remote sensing

## Abstract

Smart agriculture is a new concept that combines agriculture and new technologies to improve the yield’s quality and quantity as well as facilitate many tasks for farmers in managing orchards. An essential factor in smart agriculture is tree crown segmentation, which helps farmers automatically monitor their orchards and get information about each tree. However, one of the main problems, in this case, is when the trees are close to each other, which means that it would be difficult for the algorithm to delineate the crowns correctly. This paper used satellite images and machine learning algorithms to segment and classify trees in overlapping orchards. The data used are images from the Moroccan Mohammed VI satellite, and the study region is the OUARGHA citrus orchard located in Morocco. Our approach starts by segmenting the rows inside the parcel and finding all the trees there, getting their canopies, and classifying them by size. In general, the model inputs the parcel’s image and other field measurements to classify the trees into three classes: missing/weak, normal, or big. Finally, the results are visualized in a map containing all the trees with their classes. For the results, we obtained a score of 0.93 F-measure in rows segmentation. Additionally, several field comparisons were performed to validate the classification, dozens of trees were compared and the results were very good. This paper aims to help farmers to quickly and automatically classify trees by crown size, even if there are overlapping orchards, in order to easily monitor each tree’s health and understand the tree’s distribution in the field.

## 1. Introduction

Agriculture is one of the oldest and most fundamental human major fields. In recent years, it has become more important because it provides the basic necessities for living. Thanks to new technologies, including satellite images and artificial intelligence, agriculture is becoming smart. Many tasks have been made easier for farmers, and the yield is increasing in quality and quantity. Among the crops that can be studied are tree crops, and one of the essential components is the canopy [1,2].

The canopy is the aerial part of a plant community or the top of a crop, formed by plant crowns with emergent trees and shade trees. For forests, the canopy refers to the top layer or habitat area created by the mature tree’s crown. Knowing the crown’s size is essential for orchard management, decision-making, irrigation, fertilization, spraying, pruning, and yield estimation [3]. To obtain the tree’s crown size information from an image, it is necessary to use segmentation techniques [4]. The latter presents many challenges, such as the types of images to be used, the appropriate algorithm to considered, the technique of validating the results, etc. For example, many projects use unmanned aerial vehicles (UAV) [5], while others use high-resolution satellites images [6]. However, each of them has some advantages and disadvantages. The UAV or satellite images selection depends mainly on resolution, historical data, cost, etc. In addition, the overlap of trees is a significant factor that influences segmentation because, in most of these cases, the crowns cannot be separated from each other. For example, some citrus varieties such as mandarin and citrange have a crown diameter between three and five meters [7]. So, if the distance between two trees is less than three meters between them, they will overlap.

**Figure 1:**
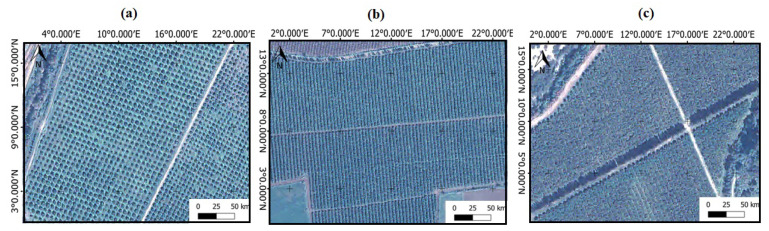
Different tree’s distribution in an orchard: (**a**) non-overlapping, (**b**) semi-overlapping, and (**c**) fully overlapping.

In general, we can distinguish three types of orchards: the first (Figure 1a) are separate trees (not overlapping), the second (Figure 1b) are trees that form rows (semi-overlapping), and third (Figure 1c), trees that are very condensed (fully overlapping).

Technically, tree canopy classification and segmentation based on remote sensing images is one of the main challenges in smart agriculture [8,9,10,11,12]. The complexity of this challenge is different from one orchard to another. It depends on the trees distribution in the orchard, the size of the canopies, and the images’ resolution. In some cases, the trees do not overlap in the field, meaning they can be directly detected and segmented using object segmentation algorithms [13,14,15,16,17]. However, sometimes the trees overlap, making it difficult for the algorithms to segment each one individually. In this case, there are other solutions, such as segmenting rows or the entire parcel canopy. Thus, these segmentations require high-resolution images to obtain good results (a minimum of 0.7 m in spatial resolution) [10,18,19,20,21,22,23].

Currently, there are a lot of approaches and techniques to achieve object segmentation. For example, some classical algorithms follow the thresholding technique by taking the image in grayscale [24]. There are clustering algorithms that follow unsupervised learning by dividing data into several groups (clusters) with common characteristics between them (similarity), based on several mathematical metrics such as Euclidean distance, Jaccard coefficient, value cosine, etc. [25]. In addition, advanced machine learning algorithms such as deep learning [26,27] train on data, which means they need several images with their ground truths (labels) to have a good result [28].

There are two tree segmentation cases in the literature: the non-overlapping orchards and the overlapping orchards. The non-overlapping orchard case has attracted the attention of many scientific researchers for a long time. In 1996, Ref. [29] tried to calculate the canopy area of tomato plants from RGB images using machine vision techniques. Their segmentation process adopted a thresholding technique, followed by an Otsu method, and extended it to an efficient multimode operation. Moreover, the canopy area determined by machine vision was compared to those measured by human operators using interactive video tracing to obtain the results. Another study [30] extracted Gabor filter texture features from remote sensing images and applied K-means [31] clustering analysis to extract the tree’s crown. After that, they used some morphological operations to reduce false detection. Their approach has a score of 0.79 F-measure. Valeriano et al. [32] tried to estimate the orange yield based on laser scanner data and the k-means algorithm. Their system used a laser scanner to obtain the point belonging to the tree’s crown.

After processing the data, they got a 3D image of each tree which was used to input the k-means algorithm. The model counts the fruits and estimates their diameter by counting the pixels of each fruit. Finally, they compared the fruit quantity obtained by the algorithm with the actual amount in the field, and they got a score of 0.88 R^2^ (coefficient of determination). Zhao et al. [33] used a UAV to collect a large dataset of pomegranate tree images and segmented the canopy using deep learning. The study was done in an orchard with non-overlapping trees, and they tested two architectures, mask-rcnn [34] and Unet [35], and got 0.98 and 0.61 (recall), respectively. To sum up, several studies attempt to segment the tree canopy in a non-overlapping orchard with good results that do not exceed an error of 10% [16,18,36,37].

On the other hand, it is challenging to distinguish each tree individually using remote sensing images in overlapping orchards. Because sometimes the trees are in rows (semi-overlapping), while in other cases, the canopy covers all the parcels, which means it is impossible to see the soil in the image (fully overlapping). Muhammad Moshiur et al. [38] used high-resolution satellite images to predict the mono yield in a semi-overlapping orchard and showed that canopy size is strongly correlated with it. In this process, the parcel’s canopy was segmented using the thresholding method to predict the yield. When only spectral information was used, a low score of 0.3 R^2^ was found, however, when the canopy size information was added, the results improved to 0.93 R^2^, which means that the segmentation was excellent and robust.

Paolo et al. [39] took a vineyard orchard and tried to segment the canopy to optimize resources, pruning, and plant health monitoring. They used UAVs to collect the multispectral vineyard images, which had several rows. In a visual comparison between some clustering algorithms such as Digital Elevation Model (DEM) [40], and K-means [41], they found that the k-means algorithm is more precise in canopy segmentation than the other algorithms. HyMap aerial hyperspectral images and light sensing and ranging (LiDAR) data were used by [42] to classify urban forest tree species. After the exhaustive pre-processing techniques, an image with a spatial resolution of three meters was obtained and used to segment the trees using the object-based image analysis algorithm (OBIA) [43], The validation was made with an ortho-image with a spatial resolution of 0.09 m, and a good segmentation was found. Afterward, Random Forest [44] and Multi-Class [45] algorithms were used to classify the tree species, and an overall accuracy of 87.0% and 88.9%, was reached, respectively.

Usually, when we have an overlapping orchard, it is challenging to identify each tree individually in the canopy. However, the trees rows or the total parcel canopy can be segmented [46,47].

To sum up, the orchard type (overlapping or non-overlapping) influences the segmentation verification and validation results. Since obtaining a scientific score in overlapping trees is difficult, most studies used visual or field comparisons to validate their models. On the other hand, in non-overlapping trees, the images can be labeled manually, and the model is trained to compare the model’s results with the labels to have a scientific score. This is why supervised learning, such as deep learning algorithms, was used [48].

So, this paper aims to present a new approach for extracting the tree’s canopy and classifying them according to their size in overlapping orchards.

## 2. Materials and Methods

### 2.1. Study Region

**Figure 2:**
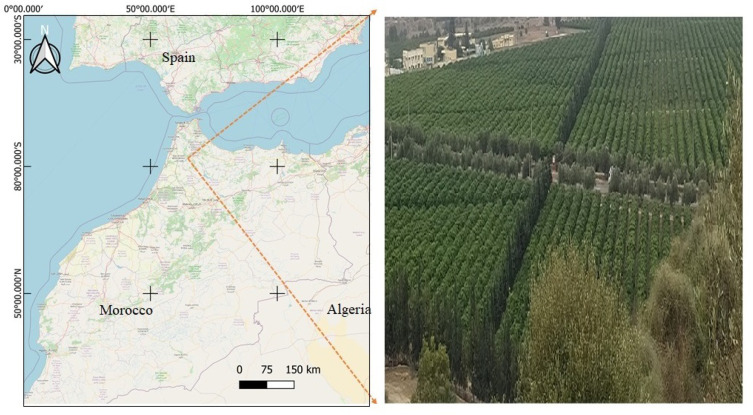
OUARGHA orchard - LES DOMAINES AGRICOLES (citrus trees).

Our studied region is Ouargha Orchard, located in Morocco, and it is the property of Les Domaines Agricoles, a Moroccan company. This orchard (Figure 2) contains citrus trees with a variety of tangerine. The trees are presented in several parcels, and the rows are clearly defined. However, the orchard is classified as semi-overlapping because it is difficult to separate each tree crown.

### 2.2. Data

The remote sensing field used images of the Earth taken by artificial satellites. In agriculture, satellite images are essential data sources. This data consists of multispectral or hyperspectral band information in several spectral resolutions [49].

In this study, we used the Moroccan Mohammed VI satellite [50], which has two satellites, A and B, launched on 8 November 2017, and 21 November 2018, respectively. One of the primary satellite objectives is to develop the agriculture field in Morocco. Mohammed VI satellite system collects multispectral images in several bands: 450–530 nm (blue), 510–590 nm (green), 620–700 nm (red), and 775–915 nm (near-infrared). This satellite is similar to the Pléiades satellite, which has a 0.5 m spatial resolution.

**Figure 3:**
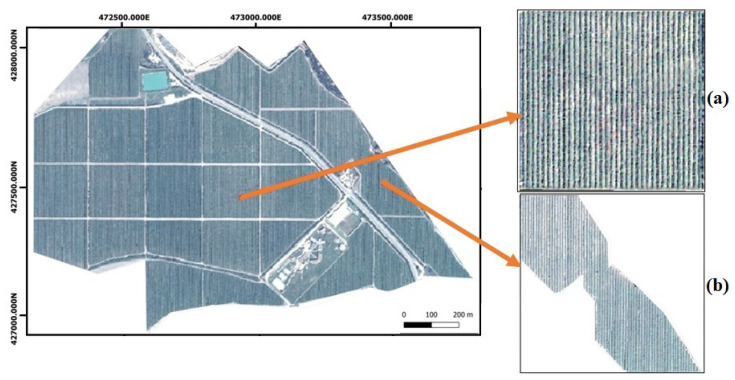
The false color (R,G,B,NIR) of the OUARGHA orchard obtained by the Mohammed VI satellite in (23 July 2020). (**a**,**b**) two examples of parcels. Projected Coordinate System: EPSG:26191—Merchich/Nord Maroc; Projection: Transverse Mercator; Linear Unit: Meter; Datum: Merchich; Prime Meridian: Greenwich; Angular Unit: Degree.

The OUARGHA orchard has 25 parcels ranging from 30,000 to 80,000 m^2^, with thousands of semi-overlapping trees. So, to carry out this research, an image captured by the Mohammed VI satellite in 2020 (Figure 3) was used and clipped by each parcel using open-source software QGIS 3.16.3 [51].

Technically we used the ’create new shapefile’ extension to create a kml file of the parcel, and we split it using the ’clip raster by mask layer’ extension.

In Figure 3, you can see an image of the study region captured by the Mohammed VI satellite.

### 2.3. Our Approach

**Figure 4:**
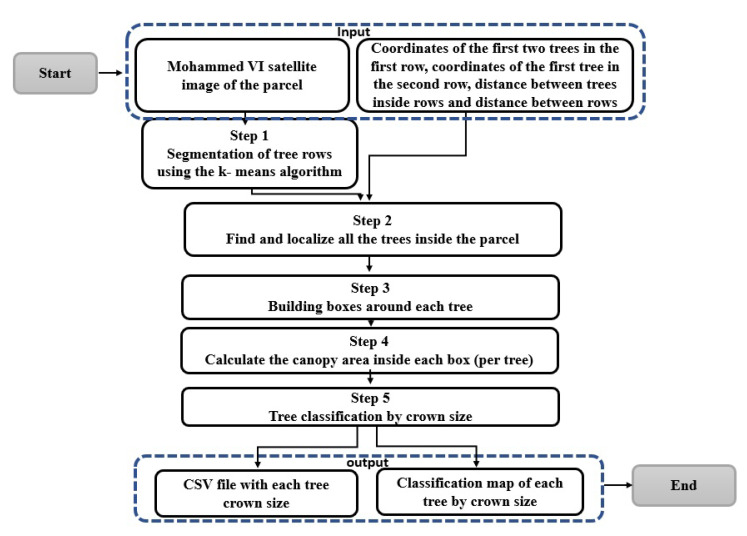
Flowchart of the methodology.

In this study, we are interested in a semi-overlapping orchard, which means that trees are presented as rows with a uniform distribution, which means that there is the same distance between all tree trunks and between rows. Our methodology used satellite images and some field measurements and consists of five steps presented in (Figure 4), which are: (1) segment the rows, (2) locate the trees, (3) create a box for each location, (4) estimate the canopy inside the boxes, and finally (5) classify these boxes according to the canopy size. Each of the steps are described in further detail.

#### 2.3.1. Tree’s Rows Segmentation

Tree segmentation is an important technique that helps farmers count their trees, get an idea about the yield based on the canopy size, and control the orchard’s health. However, this approach presents many difficulties in overlapping trees. In our study, the field contains several parcels with very condensed rows, which makes it challenging to segment each tree canopy individually. However, the rows can be identified and counted. So, we used the high spatial resolution Mohammed VI satellite images [50] to do this process. In fact, we tried two segmentation approaches using thresholding and clustering algorithms. Visually the thresholding algorithm did not get a good result, mainly because not all of the image bands were used. Otherwise, the clustering algorithms, especially k-means, presented a better result. To construct the model, we start by extracting the parcel from the original image. This technique gives each parcel an image with three pieces of information: trees, soil, and the space outside it, which is the image’s background. An example of two parcel images is presented in (Figure 3). Based on the k-means algorithm, our model takes all the image’s pixels and creates several clusters based on the K number. This k represents the number of centroids that the algorithm will develop—the cluster’s number. The elements in each of them have a small euclidean distance, which means they are very similar.

**Figure 5:**
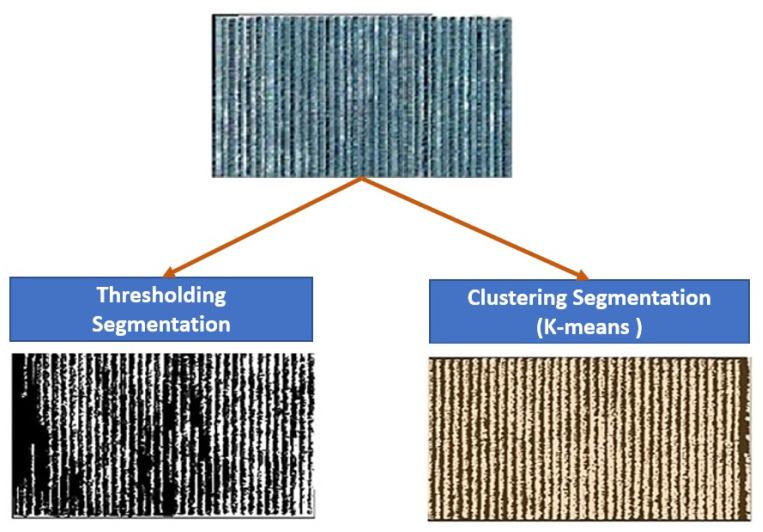
Example of tree identification using thresholding and clustering (k-means) segmentation.

In our case, we use K = 3 to get three clusters (trees, soil, and outside parcel). The results obtained are an image with one band (Figure 5). We excluded the part outside the box to get a binary mask image, with pixels of 0 and 1 values that represent the soil and the trees.

#### 2.3.2. Tree Localization

**Figure 6:**
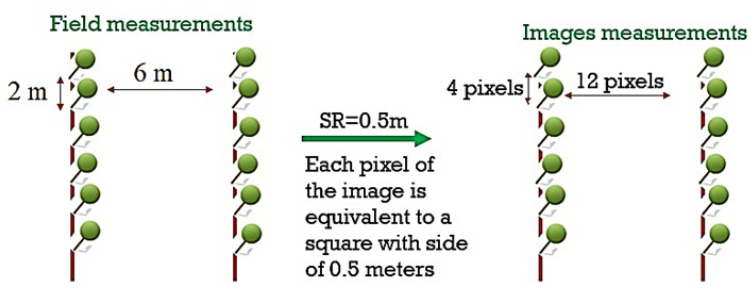
Trees distribution in the field.

In tree crops, the planting process usually follows uniform distances [52]. In our study area, the orchard has rows of trees with six meters between them and two meters between the tree trunks inside each row. In addition, the size of the canopies is heterogeneous, with large and small canopies and sometimes with weak or missing trees. The satellite spatial resolution of 50 cm means that each pixel in the image is a square with a side of half a meter. Thus, a distance of two meters between trees inside the rows equals four pixels in the image, while six-meter lengths equal 12 pixels (Figure 6).

**Figure 7:**
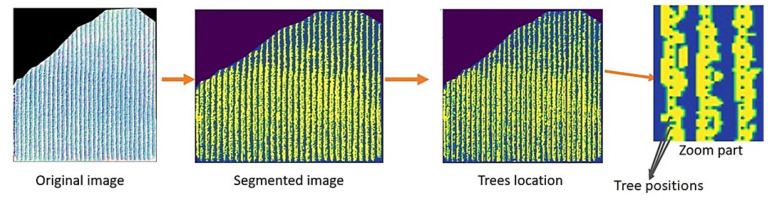
Tree localization and distribution in the parcel.

With this information, a semi-automatic algorithm was created to help us obtain the distribution of the trees’ locations in the image. The model takes three tree trunk positions as input, the first two positions of the first row and the first position of the second row, the distance between the trees inside the rows, and the distance between the rows. The algorithm loops on all pixels of the images and locates the center positions of the trees. Finally, when we compared the tree’s positions number in the image by the tree’s number in the orchard, we found that they are the same, which means that the algorithm considers all the tree’s positions in the image precisely (Figure 7).

#### 2.3.3. Tree Crown Detection

**Figure 8:**
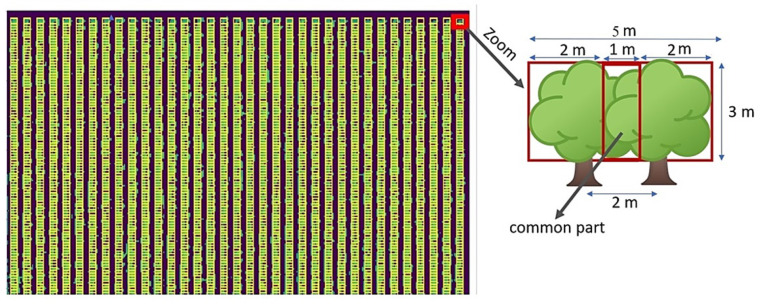
Tree crown detection using the boxes distribution in the parcel.

As shown in (Figure 5), it is difficult to separate each tree inside the rows. So, our solution was to build boxes that surround the tree’s location, and with them, get the information about each tree canopy. The first step was to find the best box sizes. In the field, trees presented a circle shape, which means a square box could be used with some caveats. In the orchard, the maximum diameter of a tree is three meters, and the distance between them is two meters. Thus, there is an overlapping of one meter between every two trees. Based on this measure, we used boxes of three meters, which have a common part between them (Figure 8).

#### 2.3.4. Tree Classification by Size

After locating the trees and creating the boxes, we moved to get the crowns by counting the pixels that describe the canopy. The model loops through all the boxes and outputs a CSV file containing each crown size and its identifier. We have created three tree classes: missing or weak, normal, and big canopies to make a classification. The canopy sizes of the trees in each one of them is calculated using the formulas follow: 

**class 1**: min_value≤canopy size<min_value+avg_value/5

**class 2**: min_value+avg_value/5≤canopy size<max_value−avg_value/5

**class 3**: max_value−avg_value/5≤canopy size≤max_value

min_value = the minimum canopy in the parcel

max_value = the maximum canopy in the parcel

avg_value = the tree’s canopies average in the parcel

## 3. Results and Validation

**Figure 9:**
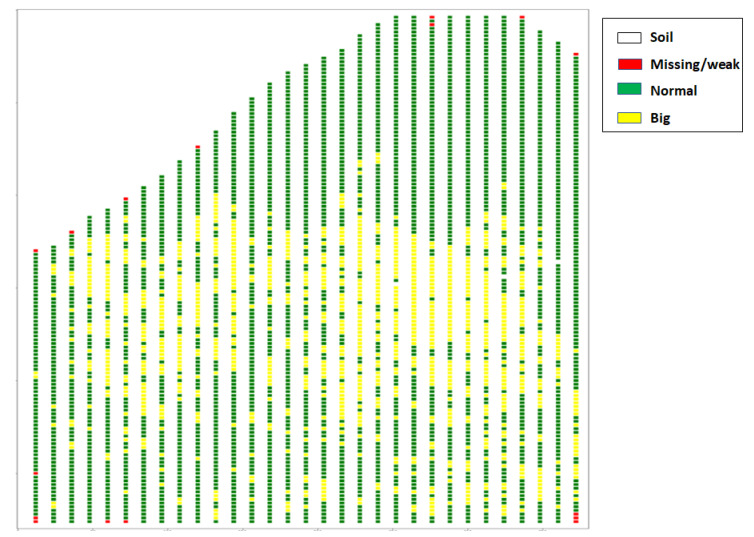
Tree classification by size in the parcel.

We took an empty image with zero in all pixels to map the classification results. Each box location was defined, so after classifying each tree, we assigned a fixed number to all the pixels of the same box in the empty image. Finally, a matrix with four pixels of value, the three classes, and the background was obtained and colored by a specific color to visualize the results as a single map (Figure 9).

**Figure 10:**
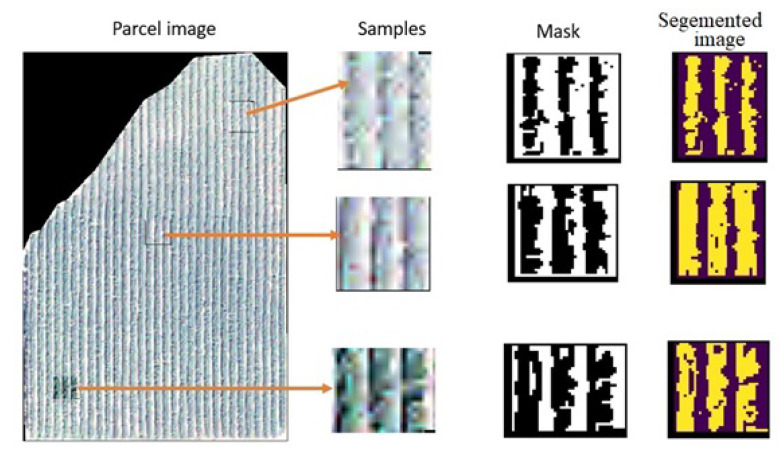
Examples of the samples, maks, and the segmented image results.

We counted the segmented rows to compare them with the absolute number of rows in the field to validate the results. After having visited all the parcels, we found the same number of rows. The next step was to validate the segmentation algorithm results. In this process, five plots with different varieties were visited in the field, with 24 of them representing 20%. Then, three samples in several positions were taken from each parcel seen in the orchards (Figure 10). Finally, we obtained 15 samples manually labeled and used them to validate the segmentation.

Finally, the results obtained by the algorithm and the mask that we manually created were compared. The metrics used were the F1 and Precision score.

(1) $$\mathrm{Precision}=\frac{\mathrm{TP}}{\mathrm{TP}+\mathrm{FP}}$$

(2) $$F1\text{-}\mathrm{score}=\frac{\mathrm{TP}}{\mathrm{TP}+\frac{1}{2}\left(\mathrm{FP}+\mathrm{FN}\right)}$$

TP = true positives;

FP = false positives;

FN = false negatives. 

The scores obtained for the five parcels are presented in (Table 1).

The results obtained during the segmentation in the five parcels were, on average, 0.93 F1-score. This part tried to compare the map obtained with the actual distribution of trees in the orchards. Indeed, it is not easy to visit all the trees and parcels because the orchard is extensive. However, we took a random tree’s sample in different parcels to validate the results.

**Figure 11:**
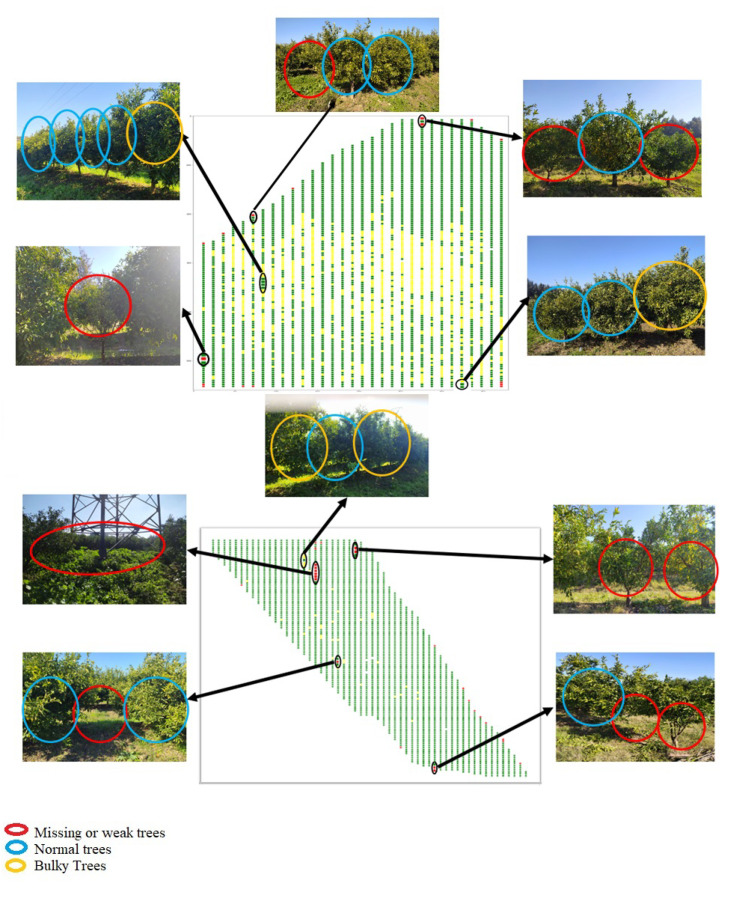
Comparison between tree classification in the map and field validation.

To validate a random tree, we located it in the resulting map with the row number to which it belongs and its order in this row to quickly facilitate their visit to the field. After visiting many trees in multiples locations with several canopy sizes, (see some examples in Figure 11), we compared the map results with the field measurements, and good accuracy in the model was found.

In (Figure 11), the blue circle means a normal tree, the yellow a large tree, and the red a missing or weak tree. It is difficult to distinguish between weak and absent trees with this approach because, in the missing tree location, the side trees find space to branch out, which means that there is a high probability that the box contains a canopy from the neighboring trees. Finally, based on this approach, farmers can easily monitor the situation of an overlapping orchard by identifying the places that contain weak, missing, and large trees.

## 4. Conclusions

Remote sensing has been used to identify tree crops without overlaps for a long time. However, when there is some tree overlapping in the orchards, a new techniques must be used. This study proposes a new approach to localize and classify trees using high-resolution satellite images and field measures in an overlapping orchard. The results were presented in a map with the distribution of the trees organized by crown size. Our methodology can be used in several other tree crops in Morocco or worldwide. However, this requires high-resolution remote sensing images, with 0.7 m at minimum, and the pattern distances between trees must be uniform. Therefore, our approach can be an alternative solution to tree monitoring problems found in overlapping orchards. For example, the vegetation mapping for each tree could be obtained by combining the map of the tree obtained by our model and the vegetation indexes obtained from the spectral information, similarly for water stress. Our approach can also provide data such as the number of trees missing, normal and big trees in each row, the total canopy of the parcel, the vegetation by tree class, etc. Finally, it is also essential to mention that this data can estimate the yield, which is a precision challenge in agriculture.

## Figures and Tables

**Table 1:** Segmentation scores.

Parcel	F1-Score	Precision
Parcel 1	0.93	0.97
Parcel 2	0.91	0.95
Parcel 3	0.93	0.96
Parcel 4	0.93	0.97
Parcel 5	0.94	0.98
